# Supplementary figures and images for: On-target inhibition of Cryptosporidium parvum by nitazoxanide (NTZ) and paclitaxel (PTX) validated using a novel MDR1-transgenic host cell model and algorithms to quantify the effect on the parasite target
Source: PLoS Negl Trop Dis. 2023 Mar 27;17(3):e0011217. doi: 10.1371/journal.pntd.0011217 (PMC10079235; doi:10.1371/journal.pntd.0011217)

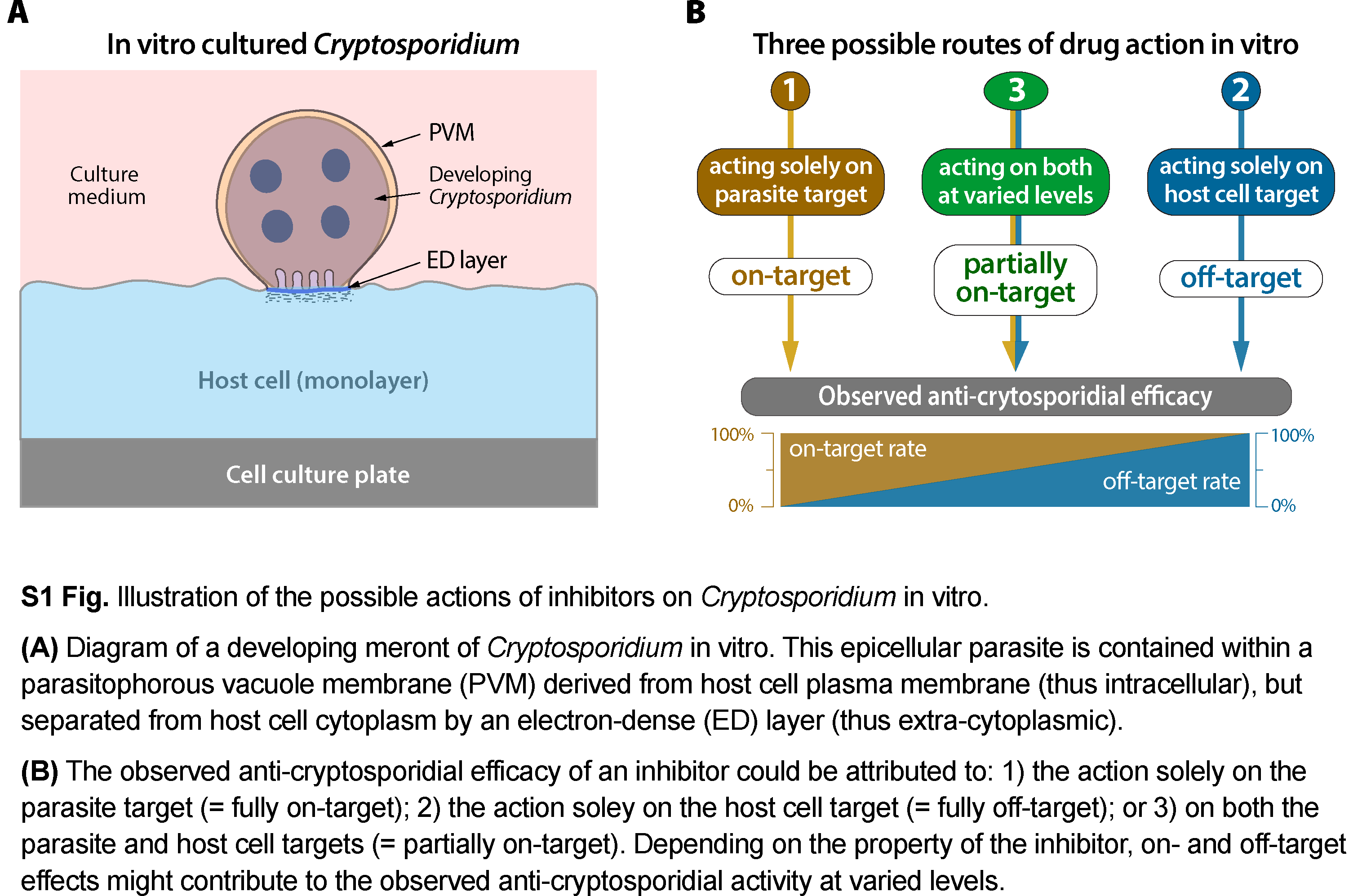

Supplement: S1 Fig — (A) Diagram of a developing meront of C. parvum in vitro. This epicellular parasite is contained within a parasitophorous vacuole membrane (PVM) derived from host cell plasma membrane (thus intracellular), but separated from host cell cytoplasm by an electron-dense (ED) layer (thus extra-cytoplasmic). (B) The observed anti-cryptosporidial efficacy of an inhibitor could be attributed to: 1) the action solely on the parasite target (= fully on-target); 2) the action soley on the host cell target (= fully off-target); or 3) on both the parasite and host cell targets (= partially on-target). Depending on the property of the inhibitor, on- and off-target effects might contribute to the observed anti-cryptosporidial activity at varied levels. (TIF) [file pntd.0011217.s001.tif]
