# Supplementary material for: On-target inhibition of Cryptosporidium parvum by nitazoxanide (NTZ) and paclitaxel (PTX) validated using a novel MDR1-transgenic host cell model and algorithms to quantify the effect on the parasite target
Source: PLoS Negl Trop Dis. 2023 Mar 27;17(3):e0011217. doi: 10.1371/journal.pntd.0011217 (PMC10079235; doi:10.1371/journal.pntd.0011217)
Supplement: Attachment — Submitted filename: #Response2reviewers_MDR1#2.docx [file pntd.0011217.s008.docx]

**Point-to-point responses to reviewers’ Comments**

We sincerely apologize for the delay in returning the revised manuscript, which is largely due to the multiple city/campus lockdowns and then a wave of infection in the lab before and after the “zero-covid” policy!

We appreciate the time and efforts from the reviewers whose comments are very helpful in improving our manuscript! The manuscript has been carefully revised in response to the comments, plus some other improvements. Below is our point-to-point responses.

**Comment #1.**

The authors developed a novel anti-cryptosporidial drug model in vitro. How to evaluate the efficacy of these drugs anti-cryptosporidial effects (kill Cryptosporidium)? The methods in this manuscript was limited, which could not effectively evaluate the anti-cryptosporidial effects. So, these results and limited should be describe in discussion part.

**Response:**

Thanks for the comment! Indeed, the new model reported here has limitations. The new in vitro model uses a set of HCT-8-based cell lines (e.g., wild-type [WT] and MDR-transgenic) to quantify the “on-the-parasite-target” effect of anti-cryptosporidial compounds. However, it cannot tell which parasite molecule(s) is targeted by the drug. Therefore, it is unable to “identify the target”. The limitations are more clearly discussed in the revised manuscript. The Methods and Discussion sections have also been revised for better clarity.

**Comment #2.**

In the cytotoxicity test, the biological function changes of HCT-8 cells should be added.

**Response:**

This is a good suggestion to include more parameters related to biological changes of host cells in cytotoxicity test. This study has provided standard cytotoxicity parameters including TC_50_ (aka. CC_50_) values on host cells for individual compounds and the effect of a compound on the host cell morphology at concentrations showing anti-cryptosporidial efficacy. These are sufficient for determining the safety intervals (aka. safety windows) and for quantifying how much an anti-cryptosporidial compound acts on the parasite target.

The suggested biological function changes are part of our follow-up research, which are ongoing. Briefly, based on the confirmation that nitazoxanide and paclitaxel “kill” Cryptosporidium by acting fully on the parasite, we are now conducting experiments to determine and verify their parasite targets, as well as to investigate how they affect the biology, including effects on the cellular and metabolic features in the parasite and the host. We will report our findings in future manuscript.

**Comment #3.**

The function of MDR1 and its function in Cryptosporidium infection should be described in background.

**Response:**

As suggested, the function of MDR1 and its function in Crypto have been more clearly stated.

**Comment #4.**

According to the methods and the results in this study, the authors validated that NTZ killed C. parvum by solely acting on the parasite target. There was limited.

**Response:**

The limitation is more clearly stated. Also see response to comment #1.

**Comments #5 and #6:**
5. Line 3: Use of the terms on-target and off-target are confusing here, and not aligned with their standard use in pharmacology. “On-target” typically refers to action on the target of the therapeutic effect and “off-target” usually refers to a toxic effect. Instead, consider “parasite-target” and “host-target”.

6. Line 3: Use of the term “rates” in the title (“on/off-target rates”) is confusing, as this term typically refers to something happening over time. It also evokes an “off-rate” in a molecular binding, which is not at all what is being referred to here. I suspect that what the authors mean is “ratio”, but I suggest using the term “effect”, as in the Short Title.

**Response:**

We agree. The suggestions are very helpful in making the title clearer! Line 3 in the title is changed to “algorithms to quantify the effect on the parasite-target”. The terms are correctly and appropriately used in the Short title, Abstract and main text.

In the main text, for simplicity and easy reading, “on-target” and “off-target” are used only after we have defined the two terms in the Introduction (last two sentences in paragraph #2): “For simplicity, hereinafter we will use “on-target” effect to describe the action of an inhibitor “on the parasite target” and “off-target” effect to describe the action of an inhibitor “off the parasite target” (i.e., on the host target).”

**Comment #7.**

7. Lines 95-100: I suggest an additional comment here about why it is important to understand MOA. For example, better understanding of MOA can enable optimization of drugs, reduction in toxic effects, and identification of new leads with the same or similar MOA.

**Response:**

Great suggestion! The statement has been added to the manuscript.

**Comment #8.**

8. Line 134: A critical hypothesis that is not at all addressed in this manuscript is that NTZ’s broad spectrum antimicrobial activity (reported against bacteria, viruses, and parasites) is due to stimulation of the host immune system, rather than a direct effect on pathogens (Jaseonsky et al. 2019 iScience DOI: 10.1016/j.isci.2019.07.003; Dang et al. 2018 Antimicrob Agents Chemother DOI: 10.1128/AAC.00707-18; Elazar et al. 2009 Gastroenterol DOI: 10.1053/j.gastro.2009.07.056; etc.). While this hypothesis does not exclude the possibility that NTZ may have direct effects on Cryptosporidium and/or direct effects on infected host cells that inhibit parasite growth, it should be acknowledged that this HCT-8/MDR1-transgenic model does not enable investigation of NTZ effects on immune response.

**Response:**

These are nice points that we had overlooked. These points are now discussed in the revised manuscript, including the addition of relevant references.

**Comments #9 and #10.**

9. Line 155: drugs are “approved” or “registered” by the US FDA, only vaccines are “licensed”

10. Line 184: “…develop drug resistance more rapidly…”? According to Table S2, all lines were selected for the same duration. Instead consider, “…develop greater drug resistance…”

**Response:**

The sentence has been corrected/reworded – thanks for the suggestions!

**Comment #11.**

11. Lines 209-210: If there is no increase in MDR1 protein expression by IFA, what is the basis of increased PXT or NTZ tolerance in selected cells?

**Response:**

This is an intriguing question to us, too. Mutations in endogenous and/or transgenic MDR1 gene is likely an explanation as demonstrated by other investigators (e.g., PMID: 32220325). This hypothesis is being tested by transcriptomic and proteomic analysis in our follow-up study, which will be reported later. In this manuscript, we added a discussion to speculate the basis of increased PXT or NTZ tolerance in selected cells.

**Comment #12.**

12. Line 360: Implying the ratio of TC50/EC50 is a major determinant of clinical drug safety could be considered misleading. Dose-limiting drug toxicity is usually not driven by cytotoxicity directly, but instead by toxic effects on other tissues (heart/QT, liver/metabolic pathways, etc.). Consider using the term “in vitro cytotoxicity index” rather than “safety interval”.

**Response:**

Nice comment! We agree that “safety interval” could be misleading and “in vitro cytotoxicity index” is more appropriate. For convenience, we are now using in vitro “selectivity index” (that is also commonly used in literature) to replace “safety interval” as both share the same abbreviation “SI”.

**Comment #13.**

13. Lines 431-441: As mentioned previously above, there should be some discussion of the potential impact of NTZ on immune modulation.

**Response:**

As suggested, discussion has been added.

**Comment #14.**

14 Line 539: Method of quantitation of western blots (phospho-imager?) in Figs 2C and 2F should be described. The bands on the westerns in Figs 2B and 2E look more than 1.5x darker/bigger.

**Response:**

The western blots used fluorescence substrate that was visualized by a gel documentation system under fluorescence mode. Band intensities were quantified using Gel Analyzer implanted in ImageJ software. The signals of GAPDH were used for normalization. The intensities looked like higher than 1.5x by naked eyes, but the normalized measurements were indeed around or slightly higher than 1.5x. The PI has also re-measured the signals and the results are very close to these presented in the manuscript.

**Comments #15 to #25:**

15. Line 44: “…newly hits/leads” should be modified to “new hits/leads” or “newly identified hits/leads”.

16. Lines 70-71: For “…big weight loss”, consider instead “significant” or “substantial”.

17. Lines 83-84: …a parasite target…a host cell target… (indefinite articles are missing for both).

18. Line 132: inhibition (not “inhibitions”)

19. Line 134: delete “for” in “This is for the first time…”

20 Line 139: allow (not “allows”)

21. Line 143: promoters

22. Line 154: It is unnecessary to spell out name of compound/assay in Results. I suggest describing this detail only in the Materials and Methods section.

23. Figure 2A: pCMV, not “pMCV”

24. Figure 7A: “inhibior” is misspelled

25. Figure 11B: “efficay” is misspelled

**Response:**

We really appreciate the reviewers' careful reading! These typos or editing issues have been corrected in the revised manuscript.
